# Detailed Emotional Profile of Secondary Education Students Toward Learning Physics and Chemistry

**DOI:** 10.3389/fpsyg.2021.659009

**Published:** 2021-08-04

**Authors:** María Antonia Dávila-Acedo, Diego Airado-Rodríguez, Florentina Cañada-Cañada, Jesús Sánchez-Martín

**Affiliations:** ^1^Department of Science and Mathematics Education, University of Extremadura, Badajoz, Spain; ^2^Department of Science Education, University of Jaén, Jaén, Spain

**Keywords:** emotions, Principal Component Analysis, pupils, Secondary Education, Physics and Chemistry, content, teacher

## Abstract

The present research arises from the need to identify the emotions that K-7 to K-10 students experience toward the learning of Physics and Chemistry, since it is a fact that there is a decrease in the number of students choosing itineraries related to Science. Different blocks of contents have been considered in each subject in order to identify emotions toward each one of them. The considered sample consisted of 149 K-8 students, 152 K-9 students and 130 K-10 students from several middle and high schools in Badajoz (Spain) during the 2014–2015 school year. Students experienced more positive emotions toward the content of Chemistry than toward those of Physics. A decrease was detected in the mean frequency of positive emotions such as joy, fun, and tranquility from K-8 to K-10, as well as an increase in negative emotions such as boredom, anxiety, disgust, fear, nervousness, worry, and sadness. It has also been found that positive emotions toward Chemistry contents are mainly related to teachers’ methods and attitudes, while negative emotions toward Physics contents are related to the exclusive use of the textbook, solving Physics problems, or giving oral presentations of the topics in class.

## Introduction

Nowadays the concept of sustainability has reached dimensions beyond the simple environmental care and includes sociological, economic, ethical, or cultural dimensions (Zamora-Polo and Sánchez-Martín, 2019). The birth of this comprehensive vision of sustainability is intimately linked with the promulgation of the Sustainable Development Goals (General Assembly of United Nations, 2015). Considering this holistic vision of sustainability, the training of students supposes a key aspect for change, in the construction and implementation of a way of understanding sustainability from the cognitive, the emotional, the civic, and the sociocultural dimensions.

Education has been traditionally situated in the center of sustainable human development, and in this regard, science education plays a key role. There are no doubts of the existence of clear interrelationships between experimental sciences and the questions of civic responsibility or of citizenship at a planetary scale. In this line, few disciplines are as closely related to the classical idea of sustainability as science teaching (Colucci-Gray et al., 2013). Scientific literacy is a must to form responsible citizens and this formation needs to be carried out in the school, making the introduction of sciences and STEM areas in general in the curriculum crucial at all educational stages.

Hargreaves (2000) stated more than 20 years ago that in the teaching-learning process the cognitive dimension is influenced by the affective one and vice versa. Numerous studies point in the direction that both cognitive and affective aspects influence the teaching and learning processes (Alsop and Watts, 2003; Hargreaves, 2003; Shapiro, 2010). Results indicate that teachers who ignore the affective aspects of learning might be limiting conceptual change in their pupils (Duit and Treagust, 2012; Chiang and Liu, 2014). Pintrich et al. (1993) had already questioned the so-called “cold change” and defended the importance of motivation and emotions as determining factors in science learning. According to Tobin (2012) and Tomas and Ritchie (2012), emotions are a central part of the action of learning science, and they act as a social glue that interconnects individual and collective interests and actions. Emotions are also linked to action, decision-making (Damasio, 2010), and academic achievement (Pekrun and Linnenbrink-García, 2014; Goetz et al., 2016), which turns especially important for pupils, when at the end of their compulsory education, they have to decide about their future studies. Knowledge of pupils’ emotions toward the science, specifically toward Physics and Chemistry, would help teachers to properly plan the teaching and learning process to make it more effective and attractive for their pupils (Cheung, 2011). Therefore, science is not sustainable if taught and learned from assumptions that do not contemplate the affective domain.

Garritz (2010) pointed out that traditionally science has been mainly represented in schools as an area of the rational, analytical curriculum, with hardly any relation to emotions. For years, social, cultural, and emotional factors have been excluded, being labeled by the dominant positivist orientations as improper or unscientific, being contrary to the objectivity of science (Alsop and Watts, 2003).

Learning science is much more than a cognitive process because, in order to learn, it is necessary to be able to do and to want to do (Bacete and Betoret, 2000). If, as pointed out by Bisquerra (2009), academic knowledge is learnt better when the pupils have emotional competencies then it is necessary to analyze both the cognitive and the affective aspects of learning different scientific content. Diagnoses of the emotions that occur every day in secondary classrooms will therefore provide a basis for intervention in the improvement of science learning by designing activities that promote more positive emotions (King et al., 2015), since positive emotions foster learning whereas negative emotions limit the ability to learn (Pekrun, 1992).

The concept of emotion has been studied in various lines of research from different perspectives. They all show that it is a complex process which analyses the subjective reactions to a situation or personal event which entails both physiological and behavioral changes (Bisquerra, 2009; Kelchtermans and Deketelaere, 2016). There are many taxonomies for the classification of emotions. Namely, focusing on their effects upon behavior (Bisquerra, 2009), two types of emotions can be distinguished: Positive and negative. Positive emotions produce pleasant feelings, with short temporal duration, and negative emotions produce unpleasant feelings and the mobilization of many resources to face them. Other authors such as Posner et al. (2005) have proposed models based on the interaction between the intensity of emotions or the level of activation of the individual (excitation/relaxation) and the assessment of the situation which involves these emotions (pleasant/unpleasant).

Nonetheless, as pointed out by Tomas and Ritchie (2012), there have still only been a few studies focused on the role of emotions in the learning of specific science content. It is therefore essential to continue in this line and to deep into the identification of the influence of emotions in the learning of the different contents of the science curriculum (Garritz, 2010).

Research shows that K-1 to K-6 pupils usually have positive emotions and attitudes toward science (Mellado et al., 2014) but that these decrease with age, especially by K-8 to K-10 (Osborne et al., 2003). During K-7 to K-10 levels, the emotions toward science depend on the content (Borrachero et al., 2014), with more positive attitudes toward Biology and Geology than to Physics and Chemistry.

K-7 to K-10 students’ positive emotions toward particular science content are related to self-efficacy, or the belief in their own ability and competence to learn that content. Self-efficacy is closely related to self-regulation, and it is a powerful variable which enables the prediction of students’ achievement (Cakiroglu et al., 2012). Borrachero et al. (2014) showed that when the students felt that they were able to learn certain content they showed an increase in their positive emotions toward that content. But when they did not feel capable of learning the content, they more often experienced negative emotions. This is especially important in Physics and Chemistry for which more negative emotions are recorded and self-efficacy has most influence (Brígido et al., 2013).

Attitudinal and emotional depression toward sciences is attributed to the fact that K-7 to K-10 students create an image of school science as boring, difficult, overly theoretical, and of little use. Other causes that might have an influence are the teacher, the lack of practical work, or the excessive orientation in the classes to preparing for examinations (Murphy and Beggs, 2003).

In any case, as acknowledged by Wan and Lee (2017), more studies are needed to deal with the analysis of the causal relationships between the cognitive and the affective dimensions in K-7 to K-10 levels when learning sciences.

## Research Objective

The present research aims to achieve the following objectives:

1. To determine and to analyze the relationship between the positive and negative emotions experienced by K-8, K-9 and K-10 students when receiving Physics and Chemistry lessons.
2. To determine and to analyze the relationship between certain aspects related to teacher and student and their possible implication as causes of positive or negative emotions toward Physics and Chemistry.

## Methodology

### Sample

The process carried out to select participants was cluster sampling. This provided a representative sample of Secondary Education (K-8 to K-10) of a city with about 150,000 inhabitants like the city of Badajoz (Spain), where the study has been carried out. Access and convenience sampling have been the implemented inclusion criteria, looking for a homogeneous distribution throughout the city.

The considered sample consisted of 431 students, according to the distribution summarized in Table 1.

**TABLE 1:** Distribution of students by level.

Level	Number of students	Percentage (%)
K-8	149	36.4
K-9	152	35.3
K-10	130	30.2

Sociodemographically the sample featured 47.1% girls and 52.9% boys with ages ranging between 13 and 17 years old. It is an urban sample, with participant students belonging to middle-class families and working parents.

### Instrument

A quantitative non-experimental or “*ex post* facto” methodological approach was considered to perform this research. The data acquisition instrument was a questionnaire of the authors’ own elaboration (Supplementary Material), based on the one previously proposed by Borrachero et al. (2014).

In the questionnaire, students are asked first about negative and positive emotions they experience when learning certain topics belonging to five blocks of contents. Those blocks of contents were established according to the current educational curriculum (*Real Decreto 83/2007*). Namely, the considered blocks of content were “Matter” (block I), “Energy and Electricity” (block II), “Structure and Changes of Matter” (block III), “Kinematics and Dynamics” (block IV) and “Work and Energy” (block V). Blocks I, II, and III were considered in K-8 to K-10, while blocks IV and V were exclusively considered for K-10 students; secondly, they are asked about the felt emotions toward several aspects related to the teacher and the students; and lastly, they are asked how often they feel seven positive and seven negative emotions when learning Physics and Chemistry. Previous research carried out by our research group allowed us to identify the selected emotions as the most representative in the academic field (Brígido et al., 2013; Borrachero et al., 2014). In all cases students are asked to respond according to an 11-points Likert scale (0 = minimum; 10 = maximum).

The reliability of the questionnaire was calculated using the covariation between the items of the different scales making it up in order to verify its internal consistency. The obtained values were all higher than 0.80, which means that the questionnaire is quite good.

### Procedure

The teachers responsible for the K-8 to K-10 levels were asked to hand out the questionnaires to the different groups of students. Data was processed and analyzed statistically using the statistical package SPSS (Statistical Product and Service Solutions) 22.0 and The Unscrambler for Windows.

A Principal Component Analysis (PCA) was performed in order to establish possible correlations between the variables, in this case content (of Physics and Chemistry) and causes (teacher/pupil).

Principal component analysis is a dimensionality reduction technique that has proven to be useful in the extraction of relevant information from complex datasets. This analysis seeks to maximize the variance of a linear combination of the variables. It maps each instance of the given dataset in a *d*-dimensional space to a *k*-dimensional subspace so that *k* < *d*. The set of *k* new dimensions are called the principal components (PC) and each principal component is directed toward a maximum variance excluding the variance already accounted for in all the preceding components. The first principal component is the linear combination with maximal variance. The second principal component is the linear combination with maximal variance in a direction orthogonal to the first principal component, etc. The first principal component also represents the line that minimizes the total sum of squared perpendicular distances from the points to the line. The principal components can be represented as:

(1) $$P\,C_{i}=a_{1}\,X_{1}+a_{2}\,X_{2}+\ldots .+a_{d}\,X_{d}$$

where:

PC*_*i*_*_:_ principal component “*i*”;

X*_*j*_*_:_ original feature “*j*”;

a*_*j*_*_:_ numerical coefficient for X*_*j*_*.

Van der Wal and Kowalczyk (2013) worked in the measurement of changes in the emotional state of a speaker by analyzing his/her voice and employed PCA to visualize the results to the speaker in the 2-d space. They reported that PCA is a promising technique for visualizing a human’s emotional state. Zhang and Zhao (2013) assayed and compared different dimensionality reduction methods, including PCA, with an aim of improving the performance on spoken emotion recognition.

PCA has been also commonly employed in the field of psychology. For instance, Gray studied the implications of psychological membership in the classroom for achievement motivation and emotions (Gray, 2017). Kroeger et al. (2017) employed PCA for the evaluation of impulsivity and emotion dysregulation in adolescents with borderline personality disorder; and Gondim et al. (2015) employed it to analyze measurements on individual differences in the regulation of emotions.

## Results and Discussion

This section describes the results obtained after performing the descriptive analysis of the emotions experienced by K-8 to K-10 students when learning Physics and Chemistry content. Results are presented according to the two proposed research objectives.

### Emotions of K-8 to K-10 Students Toward the Learning of Physics and Chemistry. Relationship Between Emotions (Positive and Negative)

**FIGURE 1:**
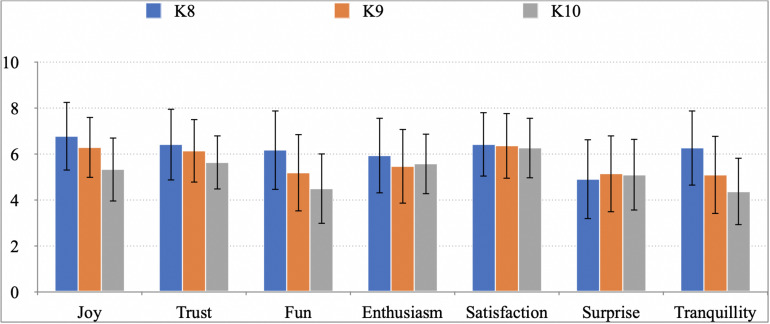
Mean frequency of positive emotions experienced by secondary pupils when learning Physics and Chemistry depending on the course.

Figure 1 shows the mean frequencies (Likert scale from 0 to 10) for positive emotions experienced by K-8 to K-10 students when learning Physics and Chemistry, depending on the level. The mean frequency ranges between 4.5 and 6.9. Maxima values are found for K-8 students, namely for emotions joy (6.78), satisfaction (6.42), and tranquility (6.26).

It can be also observed that K-8–K-10 students experienced a decrease in the average frequency of the positive emotions as joy, trust, fun, enthusiasm, and tranquility from K-8 to K-10.

A Student’s *t*-test was performed and statistically significant differences were found in the positive emotions joy (*p* = 0.000), trust (*p* = 0.045), fun (*p* = 0.000), and tranquility (*p* = 0.000) between K-8 and the other two levels, which reveals a significant decrease from K-8 to higher levels.

**FIGURE 2:**
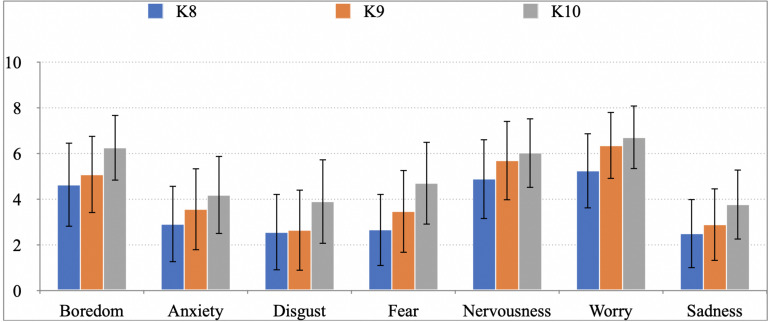
Mean frequency of negative emotions experienced by secondary pupils when learning Physics and Chemistry depending on the course.

Figure 2 shows the mean frequencies (Likert scale from 0 to 10) for negative emotions experienced by K-8–K-10 students when learning Physics and Chemistry, depending on the level. In this case, the mean frequency for negative emotions ranges between 3 and 7, with maxima in K-10 for boredom (6.25), nervousness (6.02), and worry (6.71). This might be due to the higher amount of Physics contents in K-10, whereas more Chemistry is taught in K-9.

Also, it can be observed that negative emotions such as boredom, anxiety, disgust, fear, nervousness, worry, and sadness increase in the average frequency from K-8 to K-10.

Statistical analysis of the mean frequencies reveals significant differences in boredom (*p* = 0.000), anxiety (*p* = 0.010), disgust (*p* = 0.002), fear (*p* = 0.000), nervousness (*p* = 0.014), worry (*p* = 0.000), and sadness (*p* = 0.003) between K-10 and the other two levels.

Results were also analyzed by PCA. Firstly, the KMO (Kaiser-Mayer-Olkin) index was calculated and the Bartlett’s sphericity test was applied in order to check the suitability of the considered sample (*N* = 431) to be analyzed by PCA. When applying Bartlett’s sphericity test a *p*-value < 0.05 is obtained and a high (≈1) KMO index is calculated, which means that PCA can be efficiently performed in the current dataset, i.e., fourteen emotions measured in three levels K-8–K-10.

**FIGURE 3:**
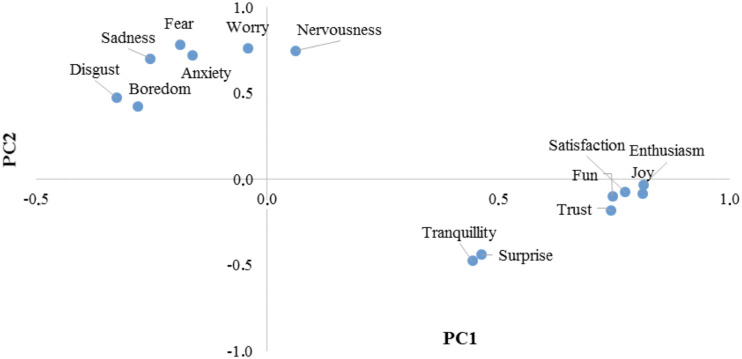
Results of the PCA on emotions of K-8 to K-10 students toward the learning of Physics and Chemistry: loadings plot for PC1 and PC2.

**FIGURE 4:**
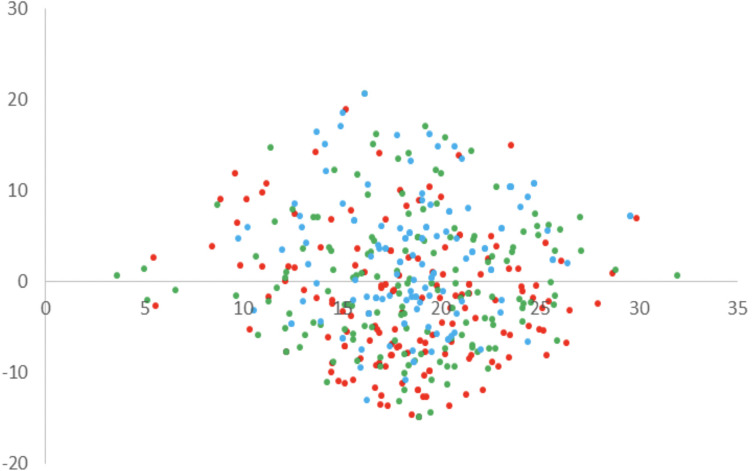
Results of the PCA on emotions of K-8 to K-10 students toward the learning of Physics and Chemistry: scores plot for PC1 and PC2 (red points: K-8; green points: K-9; blue points: K-10).

Results of PCA are summarized in Figures 3, 4 in the form of loadings and scores plots, respectively, and subsequently discussed.

Figure 3 shows the loadings plot for the plane formed by the first (PC1) and the second (PC2) principal components, which as stated above, explain 35.97 and 18.51% of total variance, respectively. This plot helps to identify correlations among variables. As it can be observed, the emotions enthusiasm, joy, satisfaction, fun, and trust present high loading values for PC1, which means that this PC represents positive emotions. It can also be observed that in the in the plane formed by PC1 and PC2 these emotions are located close to each other, which means that they are positively correlated. Nevertheless, the emotions tranquility and surprise are located apart from the other positive emotions in the plane formed by PC1 and PC2, showing lower loading values for PC1. The small loading values for emotions tranquility and surprise suggest that these emotions might not be further considered in the interpretation of the results, since they contribute in a small extension in PC1 and PC2. On the other hand, the emotions fear, worry, nervousness, sadness, and anxiety present high values of loading for PC2 and they are located close to each other in the plane formed by PC1 and PC2, which means that PC2 represents negative emotions and that the emotions above listed are positively correlated among them. The emotions boredom and disgust are located a bit farther from the cluster formed by the other negative emotions. However, since these two emotions present lower loading values for PC2, it can be concluded that the are not very important in the interpretation of the results. In the plot it can be also observed that both groups of emotions are negatively correlated.

The scores plot for the plane formed by PC1 and PC2 is represented in Figure 4. This plot helps in the identification of sample clusters and it helps also to visualize how different are sample constituents among them. In this case, as it can be observed, there is no a clear separation between students of the different levels.

To sum up, a decrease has been detected in the mean frequency of all the positive emotions and an increase in all the negative emotions from K-8 to K-10. These results can be compared to the ones obtained by Dávila et al. (2016) with a similar sample in which a decrease in the frequency of positive emotions (joy, confidence, happiness, tranquility, surprise, and excitement), and an increase in the negative emotions (worry, shame, disgust, and anger) was detected from K-9 to K-10.

The decrease in the positive and increase in the negative emotions in middle and high school in these subjects agrees with the decline of positive attitudes toward science stated in previous studies (Osborne et al., 2003). The combination of negative attitudes and emotions toward Physics and Chemistry content can influence the choice of subsequent career paths and university degrees that involve these subjects (Custodio et al., 2013) since emotions are fundamental in decision making (Angie et al., 2011). The decrease, observed in many countries, in the number of students pursuing Chemistry degrees, and even more in the case of Physics degrees, might be related to the emotionally difficult context surrounding science learning during their secondary education, when they did not manage to enjoy learning these subjects (Vázquez and Manassero, 2008).

Finally, unlike other studies (Borrachero et al., 2014), in the present one, differences according the gender were not found for any of the considered dimensions.

### Relationship Between the Considered Aspects Related to Teacher and Students With Positive Emotions

**FIGURE 5:**
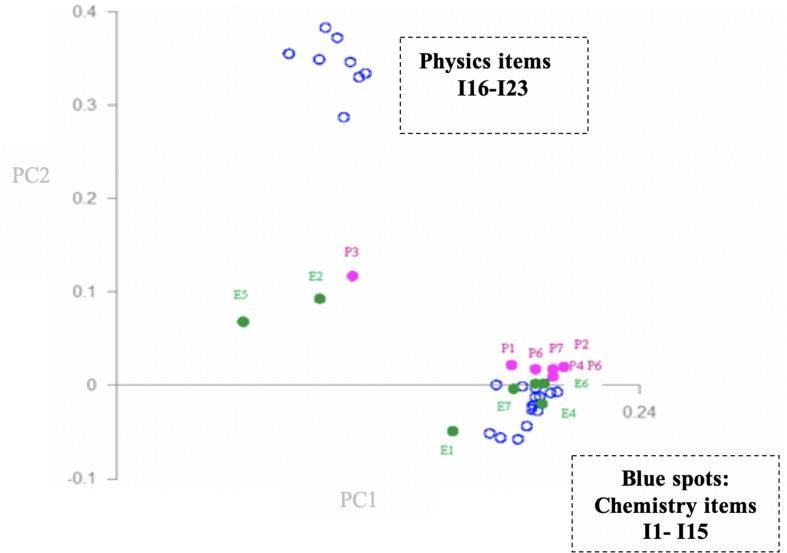
Results of the PCA on considered aspects related to teacher and students and positive emotions: Loadings plot for the principal components 1 and 2.

The part of the dataset containing measures of positive emotions toward Physics and Chemistry content, as well as toward the causes related to the teacher’s evaluation, attitude, and methods, and toward aspects related to the students, such as their ability to learn, motivation to learn, and grades obtained, was also analyzed by PCA. Figure 5 shows the loadings for the first two principal components (PC1 vs. PC2), which explain 76 and 6% of the total variance of the system, respectively. As stated above, the loadings plot resulting from a PCA is particularly useful to detect correlations between variables. Those variables with a high loading on a particular principal component (PC) define the meaning of that PC. To determine the correlation between variables, one must bear in mind that when two variables have high loadings on the same PC, those variables are strongly correlated. If the loadings of the two variables have the same sign then the correlation is positive, and negative if they have different signs.

Considering only the distribution of the various items of the content on the loadings map, it is observed that items 1–15 are grouped in the high part of the first principal component (PC1, horizontal axis), whereas items 16–23 are grouped in the high part of the second principal component (PC2, vertical axis). As items 1–15 are Chemistry content, and items 16–23 correspond to Physics content, the first conclusion that could be reached, given these variables grouping, is that the two disciplines cause in the students different quantities and types of positive emotions, which would contribute to the decoupling of the two disciplines with regard to the students’ emotions. It can also be said that all the Physics items considered in the study are strongly correlated, with this correlation being positive as they were all grouped in the high part of PC2, and that all the Chemistry items were also correlated positively with each other, being grouped in the high part of PC1. Thus, for instance, the students who show positive emotions toward the item “Atoms and molecules” (I1) also show them to the items “The periodic table and periodic properties of the elements” (I11), “Formulation and nomenclature” (I12), and “Chemical reactions and stoichiometry” (I13). Considering not only the items but also the distribution of the various causes studied related with the teacher (pink dots), one observes that all these causes except P3 (“Exclusive use of the textbook”) are in the high part of PC1, very close to the cluster formed by the Chemistry items. This means that these causes of positive emotions related to the teacher are correlated positively with the Chemistry items. For example, a pupil showing positive emotions toward Chemistry items will also show positive emotions toward aspects related with the teacher’s methods, such as doing practical laboratory activities (P1), group work and activities outside the classroom (P2), as well as to aspects related with the teacher’s attitude, such as clarification and resolution of doubts (P6), and the use of new technologies (ICT) (P7). It is logical that pupils with positive emotions toward lab work or activities outside the classroom usually do not show such emotions toward the exclusive use of textbooks (P3).

Considering also the causes related to the students (green dots), one observes that all of them except two are located within or very near the cluster formed by the Chemistry content. The two exceptions are E2 (“Giving oral presentations in class”) and E5 (“Solving physics problems”). It is logical that solving Physics problems correlates with neither the Chemistry content nor oral presentations since this is something scarcely worked during this educational stage. Again, this means that all the considered causes related to students which have been considered in this study except E2 and E5 are positively correlated with the Chemistry content as far as positive emotions are concerned. In view of these results, it can be affirmed that a pupil with positive emotions toward the Chemistry content also shows them toward aspects related to motivation and the capacity to learn, such as relating the content to daily life (E3), using diagrams to understand the content (E7), and participating in science-related debates (E4). As shown in the plot, the correlation of E1 (“Marks obtained”) with the Chemistry content is weaker than that of E3, E4, E6, and E7, probably because facing an evaluation creates uncertainty and fear in the students, regardless of the subject. Regarding the Physics items in this study, no clear correlations were observed with any of the considered aspects related to the teacher or to the students.

**FIGURE 6:**
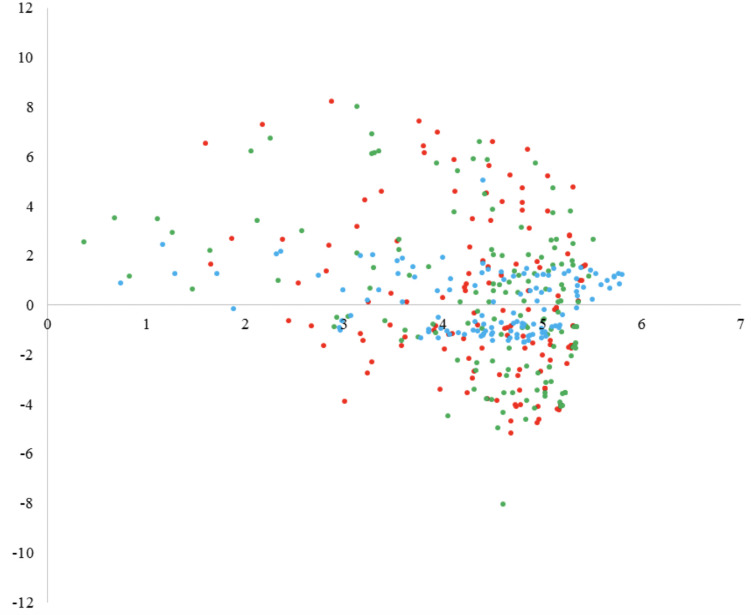
Results of the PCA on considered aspects related to teacher and students and positive emotions: Score plot for the principal components 1 and 2 (red points: K-8; green points: K-9; blue points: K-10).

The scores plot of the performed PCA on positive emotions and aspects related to teacher and students is represented in Figure 6. The score of a sample on a particular PC describes the characteristics of that sample for the variables with high loadings on that PC. Thus, samples with similar scores on the same PC can be said to be similar with respect to the variables that most contribute to that PC. The score plot is typically used to detect groupings, similarities, and differences between samples.

As it can be observed in Figure 6 most of the samples are clustered along the high part of the horizontal axis (PC1), as Chemistry topics and the causes related to the teacher and the students commented above. This means that most students expressed positive emotions toward the Chemistry content and toward the teacher- and pupil-related causes correlated with that content. But, since the maximum density of points is located in this part of the plot, one can also state that most of the positive emotions expressed by the pupils were toward the content of Chemistry, not Physics, because the density of points at the high part of PC2 is very low.

It is reported in the literature that emotions toward learning content are related to the strategies and activities undertaken in class. King et al. (2015) found that, when classes include stimulating activities and experiments about energy, the students’ emotions were very positive: Amazement, surprise, joy, and happiness. In a study with secondary pupils on physics content, Folashade and Akinbobola (2009) found that the use of methods which encourage the pupils’ active participation, such as a problem-based learning technique, leads to a better understanding of physics concepts as well as increasing the pupils’ confidence, especially for those who show low capacities for learning that content.

In the frame of the current research, internal and external causes could be related to the considered students’ and the teachers’ aspect, respectively. However, the failure attributed to internal causes led to lower self-esteem than the failure attributed to external causes. In the current research, the pupils attributed their negative emotions toward physics content to the use of the textbook in class. In contrast, they attributed positive emotions to themselves when solving a physics problem or giving oral presentations. In the case of chemistry content, the pupils who experienced positive emotions attribute them to aspects related to their ability and motivation to learn. The negative emotions, however, were attributed to the teacher’s methods and attitude.

## Conclusion and Implications

As the first conclusion of the current study it can be stated that teachers could design and use different teaching strategies so that their pupils could participate actively to interact in their learning and thus promote the development of positive emotions. In the following paragraphs, it is given a synthesis of the reached main conclusions based on the two research objectives initially posed for the study.

Regarding the first objective, statistically significant differences were found in the mean frequency of the emotions (both positive and negative) experienced by students according to their level.

Thus, in learning Physics and Chemistry, K-8–K-10 students experienced a decrease in the mean frequency of positive emotions such as joy, fun, and tranquility from K-8 to K-10. At the same time, there is an increase in the mean frequency of the negative emotions such as boredom, disgust, fear, and sadness from K-8 to K-10. This is very worrying for science learning, since K-10 is crucial for many students as it is the end of compulsory education in the Spanish system.

Regarding the second objective, one can state that there is a correlation between the topics of the Chemistry content and aspects related to the teacher’s methods and attitude, such as performing practical laboratory activities, group work and activities outside the classroom, clarification and resolution of doubts, and the use of new technologies, as well as aspects related to the pupils’ motivation and ability to learn, especially considering the relation and usefulness of the content with everyday life, the use of diagrams to understand and learn the topics of the content, and their memorization.

In addition, there is a correlation between the topics of the Physics content and aspects related to the teacher’s methods such as the exclusive use of the textbook, as well as aspects related to the pupils’ motivation and capacity to learn, such as problem solving and giving oral presentations.

Therefore, these negative emotions are not only due to the content or topic, but might also correspond to the teachers themselves or the way they teach (Daschmann et al., 2014). Sometimes teachers use the same methods or models with which they themselves were taught, focusing exclusively on the transmission of knowledge, and taking no notice of the emotional aspects that should be considered in the process of teaching and learning.

Regarding the implications, it is necessary to study the affective domain in the subject of Physics and Chemistry, and to foster the development of positive attitudes by promoting favorable feelings and emotions in order to improve students’ expectations toward this subject. It is necessary to generate positive emotions toward the teaching and learning of Physics and Chemistry, and to address the negative emotions acquired during the years at school (Mellado et al., 2014). Therefore, it is also necessary for teachers to be able to detect these emotions in their everyday work in class (Rahayu, 2015; King et al., 2017), and the use of different teaching strategies for their pupils to actively participate and interact in their learning, emerges as a must, so that students are able to appreciate the usefulness of the content of Physics and Chemistry in their daily lives.

Many studies on emotions reported in the literature are focused in the transition between primary and secondary education and most of these studies are made with university students and therefore these studies are based in the memory (Brígido et al., 2013; Borrachero et al., 2014). In this article we present a novel study which is pioneer in the fact that emotions and their causes have been monitored *in situ*, in the secondary education classroom.

Teachers are crucial in generating emotionally positive environments (Olitshy and Milne, 2012) because, as Kangas et al. (2017) noted, the success of the learning environment during secondary education, in terms of the student’s satisfaction, depends largely on the teacher’s commitment and teaching decisions. Therefore, teachers have to be aware of their own emotions, as these can have an impact on the pupils themselves (Melo et al., 2017).

It is a fact that STEM areas in general are not popular among secondary students, and it is causing a decline in scientific vocations and even an insufficient scientific literacy in the population. To solve the problem, it is necessary in first place to look for the cause. With the approach presented in this article it is shown that the methodology employed by teachers constitutes a main cause of negative emotions toward the considered disciplines. The identification of the methodology as a cause of negative emotions is in a way good news, since it implies that the solution to the problems and changing the situation is in hands of the teachers. It is stated in the literature that the implementation of innovative, motivating, and collaborative activities in secondary education science classes increases motivation, interest, and positive emotions (Ritchie et al., 2011; Tomas and Ritchie, 2012). Our research group has demonstrated, for instance, how the implementation of active, motivating, and innovative methodologies improves attitude and emotions toward scientific disciplines and definitely it is demonstrated that emotions are implied in what the student learns (Sánchez-Martín et al., 2020; Hernández-Barco et al., 2021; Yllana et al., 2021).

Future research will be to design an intervention program based on the development of practical classroom activities associated with specific content. The aim will be to improve the cognitive and emotional components of learning by motivating K-8–K-10 students in their science classes, creating confidence, helping them to understand the content, using technological resources, arousing their interest, and, in combining individual and cooperative work, favoring teacher-student and student-student interaction (Laukenmann et al., 2010; Bellocchi et al., 2013; Bellocchi and Ritchie, 2015; King et al., 2017; Sánchez-Martín et al., 2017; Jeong et al., 2019). In the future, it would be also interesting to consider new sampling strategies that allow continuous, instead of punctual, monitorization of emotions. Finally, the generation of qualitative data based on an adapted version of the elaborated questionnaires and even by means of semi-structured interviews constitutes a natural path of continuity of the presented research.

## Data Availability Statement

The raw data supporting the conclusions of this article will be made available by the authors, without undue reservation.

## Ethics Statement

The studies involving human participants were reviewed and approved by Commission of Bioethics and Biosecurity of the University of Extremadura. Written informed consent from the participants’ legal guardian/next of kin was not required to participate in this study in accordance with the national legislation and the institutional requirements.

## Author Contributions

All authors listed have made a substantial, direct and intellectual contribution to the work, and approved it for publication.

## Conflict of Interest

The authors declare that the research was conducted in the absence of any commercial or financial relationships that could be construed as a potential conflict of interest.

## Publisher’s Note

All claims expressed in this article are solely those of the authors and do not necessarily represent those of their affiliated organizations, or those of the publisher, the editors and the reviewers. Any product that may be evaluated in this article, or claim that may be made by its manufacturer, is not guaranteed or endorsed by the publisher.
