# Supplementary material for: Detailed Emotional Profile of Secondary Education Students Toward Learning Physics and Chemistry
Source: Front Psychol. 2021 Aug 4;12:659009. doi: 10.3389/fpsyg.2021.659009 (PMC8371044; doi:10.3389/fpsyg.2021.659009)
Supplement: Supplementary file 1 [file Table_1.DOCX]

**QUESTIONNAIRE ON THE EMOTIONS OF THE STUDENTS ON OF COMPULSORY SECONDARY EDUCATION**

**University of Extremadura**

**Department of Science and Mathematics education**

The questionnaire presented aims to analyze several factors related to the emotions in the students of Compulsory Secondary Education in the subjects of Science of Nature and Physics and Chemistry.

This questionnaire is completely anonymous, so it is appreciated that it is answered with sincerity.

| **Student’s data** | |
| --- | --- |
| **Sex:** Boy Girl | **Age:** |
| **Are you a repeating student?** Yes No | |
| **Your school is:** Public “Concertado” | |
| **Group:** Bilingual No bilingual | |
| **Final score in your previous course of Physics and Chemistry:** | |
| **Score in the current course of Physics and Chemistry:** | |
| **How much do you like the subject Physics and Chemistry? (0 minimum, 10 maximum)**  0 1 2 3 4 5 6 7 8 9 10 | |
| **How much do you strive to study? (0 minimum, 10 maximum)**  0 1 2 3 4 5 6 7 8 9 10 | |

1. **On the following blocks of content, rate from 0 to 10 how positive or negative emotions you feel when learning the following topics. (0 = “minimum score”; 10 = “maximum score”).**

|  |  | **Positive Emotions**  **(from 0 to 10)** | **Negative Emotions**  **(from 0 to 10)** |
| --- | --- | --- | --- |
| **Block I.**  **Matter** | Atoms and molecules |  |  |
|  | Calculation of densities of liquids and solids |  |  |
|  | Separation of the elements of a mixture |  |  |
|  | States of matter |  |  |
|  | Pure substances and mixtures |  |  |
| **Block II.**  **Energy and Electricity** | Energy and forms of energy |  |  |
|  | Heat and temperature |  |  |
|  | The changes of state of matter |  |  |
|  | Electricity; electrical circuits |  |  |
|  | Light and sound |  |  |
| **Block III.**  **Structure and Change of Matter** | The periodic table and periodic properties of the elements |  |  |
|  | Chemical formulation and nomenclature |  |  |
|  | Chemical reactions and stoichiometry |  |  |
|  | Moles, Avogadro's number, expression of concentration (molarity, %weight, %volume) |  |  |
|  | The behavior of gases: Boyle's law, Gay-Lussac's law, Charles's law |  |  |

| **Block IV.**  **Kinematics and Dynamics** | Study of motions |  |  |
| --- | --- | --- | --- |
|  | Space, velocity, and acceleration |  |  |
|  | Forces (interactions between bodies) |  |  |
|  | The principles of dynamics |  |  |
|  | Fluid force and pressure |  |  |
| **Block V.**  **Work and Energy** | Kinetic and potential energy |  |  |
|  | Work and power |  |  |
|  | Heat, effects of heat on bodies |  |  |

1. **On the following aspects related to the teacher and the student, rate from 0 to 10 those aspects that caused you positive emotions and negative emotions. (0 = “minimun score”; 10 = “maximun score”).**

|  | **Positive emotions**  **(from 0 to 10)** | **Negative emotions**  **(from 0 to 10)** |
| --- | --- | --- |
| When I do activities in the lab, I feel... |  |  |
| When the teacher uses new technologies in class, I feel like... |  |  |
| When I have to solve a physics and chemistry problem, I feel like... |  |  |
| When the teacher evaluates me, I feel... |  |  |
| When I make oral presentations or science projects in the classroom, I feel... |  |  |
| When the teacher clarifies and resolves the doubts raised in class, I feel... |  |  |
| When the contents of physics and chemistry are useful and I relate them to everyday life, I feel... |  |  |
| When the teacher uses only the textbook to teach the classes, I feel that… |  |  |
| The memorization of the contents taught in class makes me feel... |  |  |
| When the teacher proposes group activities outside the classroom, I feel like... |  |  |
| When I participate in science discussions in the classroom, I feel... |  |  |
| When the teacher motivates and encourages participation in the classes, I feel... |  |  |
| The results obtained (previous year) in the course make me feel... |  |  |
| When I use schemes to understand the contents of physics and chemistry, I feel... |  |  |

1. **Rate from 0 to 10 how often you felt or experienced the following emotions in the learning of Physics and Chemistry. (0 = Never; 10 = Maximum Frequency).**

| **EMOTIONS** | **Frequency**  **(from 0 to 10)** | **EMOTIONS** | **Frequency**  **(from 0 to 10)** |
| --- | --- | --- | --- |
| **Joy** |  | **Fear** |  |
| **Worry** |  | **Tranquility** |  |
| **Trust** |  | **Nervousness** |  |
| **Anxiety** |  | **Fun** |  |
| **Satisfaction** |  | **Disgust** |  |
| **Sadness** |  | **Enthusiasm** |  |
| **Surprise** |  | **Boredom** |  |
